# Sucking of human blood by *Placobdella costata* (O. F. Müller, 1846) (Hirudinida: Glossiphoniidae): Case study with notes on body form

**DOI:** 10.1002/ece3.8261

**Published:** 2021-12-08

**Authors:** Joanna M. Cichocka, Aleksander Bielecki, Izabela Jabłońska‐Barna, Łukasz Krajewski, Katarzyna Topolska, Joanna Hildebrand, Małgorzata Dmitryjuk, Anna Biedunkiewicz, Andrei Abramchuk

**Affiliations:** ^1^ Department of Zoology Faculty of Biology and Biotechnology University of Warmia and Mazury in Olsztyn Olsztyn Poland; ^2^ XI High School in Olsztyn Olsztyn Poland; ^3^ Department of Tourism, Recreation and Ecology Faculty of Geoengineering University of Warmia and Mazury in Olsztyn Olsztyn Poland; ^4^ Institute of Technology and Life Sciences—National Research Institute Raszyn Poland; ^5^ Wyspiańskiego 30/40 Jelenia Góra Poland; ^6^ Department of Parasitology Institute of Genetics and Microbiology University of Wrocław Wrocław Poland; ^7^ Department of Biochemistry Faculty of Biology and Biotechnology University of Warmia and Mazury Olsztyn Poland; ^8^ Department of Microbiology and Mycology Faculty of Biology and Biotechnology University of Warmia and Mazury in Olsztyn Olsztyn Poland; ^9^ Brest Regional Branch of APB—Birdlife Belarus Brest Belarus

**Keywords:** blood‐sucking leeches, host‐parasite interactions, morphometry, Rhynchobdellida, turtle leech

## Abstract

1. Four events of *Placobdella costata* sucking human blood are described.
2. Human blood was sucked by both adult and juvenile specimens of *P*. *costata*.
3. The feeding strategies of juveniles under parental care are presented.
4. New data on juvenile specimens' body form are presented.
5. Information on the potential role of mammals in dispersion and habitat preferences of leeches *P*. *costata* is considered.

## Graphical abstract

In our paper, four events of blood sucking on human by juvenile and adult specimens of *Placobdella costata* were described. We presented with a new data of juvenile specimens' body form and considered information about the potential role of mammals as hosts and dispersion vector of this leech.

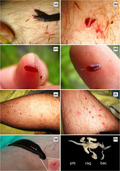

## 1 INTRODUCTION

Leeches from the genus *Placobdella* belong to the monophyletic group Rhynchobdellida (Phillips et al., 2019) and the family Glossiphoniidae, whose representatives use a muscular proboscis to collecting blood and hemolymph from subcutaneous tissues and to capture smaller victims eaten mostly whole (Bielecki et al., 2012; Borda & Siddall, 2004a, 2004b; Siddall et al., 2006). This genus with 29 species (Moser et al., 2014; Richardson et al., 2017) are primarily parasites of turtles, but amphibians, reptiles, birds, and mammals are also potential hosts (Grosser, 1996; Sawyer, 1986). This genus is widespread in North America and represented by two species in Europe—*Placobdella costata* (Siddall et al., 2005) and *P*. *ornata* (Verrill, 1872) (de Carle et al., 2017; Soors et al., 2015).

The presence of both species in Europe is closely related to the appearance of turtles, their primary hosts (Bielecki et al., 2012; Moser et al., 2016; Soors et al., 2015). The *Placobdella costata* feeds mostly on the blood of the European pond turtle *Emys orbicularis* originating from North America (Bielecki et al., 2012; Sket & Trontelj, 2008). It has also been found in the Mediterranean pond turtle *Mauremys leprosa* (El‐Mustapha et al., 2020; Romero et al., 2014), Caspian turtle *Mauremys caspica* (Bashirichelkasari & Yadollahvandmiandoab, 2017; Bielecki et al., 2012), and the Sicilian endemic pond turtle *Emys trinacris* (Arizza et al., 2016; Marrone et al., 2016). The individuals of this species are also present in regions where there are no freshwater turtles (Bielecki et al., 2012; Elliott & Tullett, 1982) and may optionally feed on the blood of birds and mammals (Bielecki et al., 2012; Elliott & Tullett, 1982; Grosser, 1996). Pawłowski (1968) claimed that leeches *P*. *costata* also attack people wading in water. Additionally, Wilkialis (1984) observed in laboratory conditions how this leech fed on human blood only in places where the skin had previously broken. Even though other species of the genus *Placobdella* (*P*. *ornata*, *P*. *rugosa*) are described as being capable of taking human blood (Klemm, 1991, 1995; Moore, 1964; Moser, 1991; Sawyer, 1972, 1986), Mandal et al. (2018) reported the first recorded observation of glossiphoniid *P*. *devkuntai sucking* human blood.

Despite having much information about *P*. *costata* and its hosts, we do not know if only adult specimens or also young leeches collect human blood and to what extent their digestive tract is filled. Furthermore, there has also been no documentation showing the noticeable negative (external) effects of blood sucking of these leeches on humans.

The aim of the paper is to present documented events of human blood sucking in natural conditions by adult *P*. *costata* leeches not caring for offspring and above all by young individuals in their care. We also described the morphological features based on the parameters of the leech body form model to show that it changes during ontogenesis.

## 2 MATERIALS AND METHODS

**FIGURE 1:**
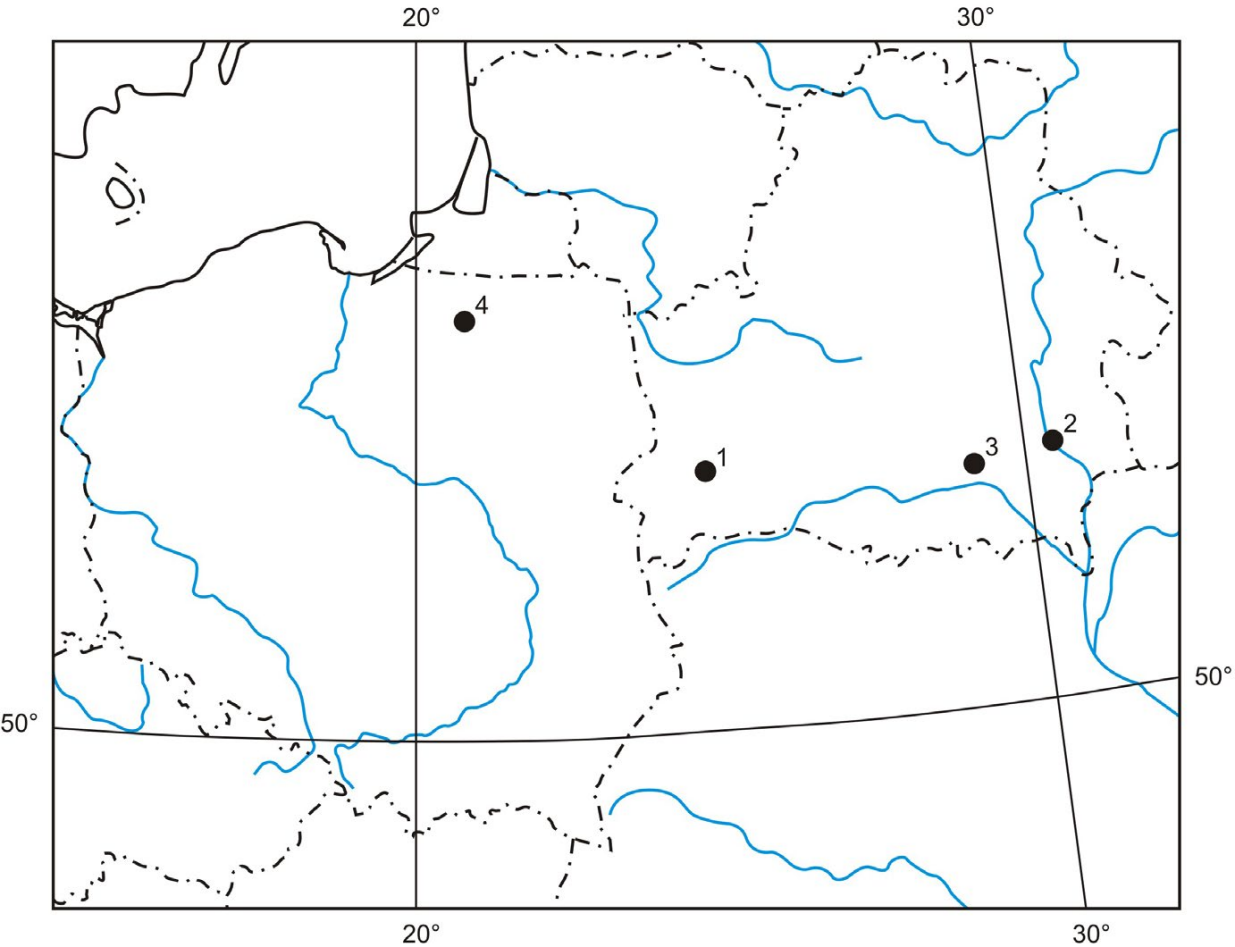
Place of events location—site numbers according to the description in Table 1

**FIGURE 2:**
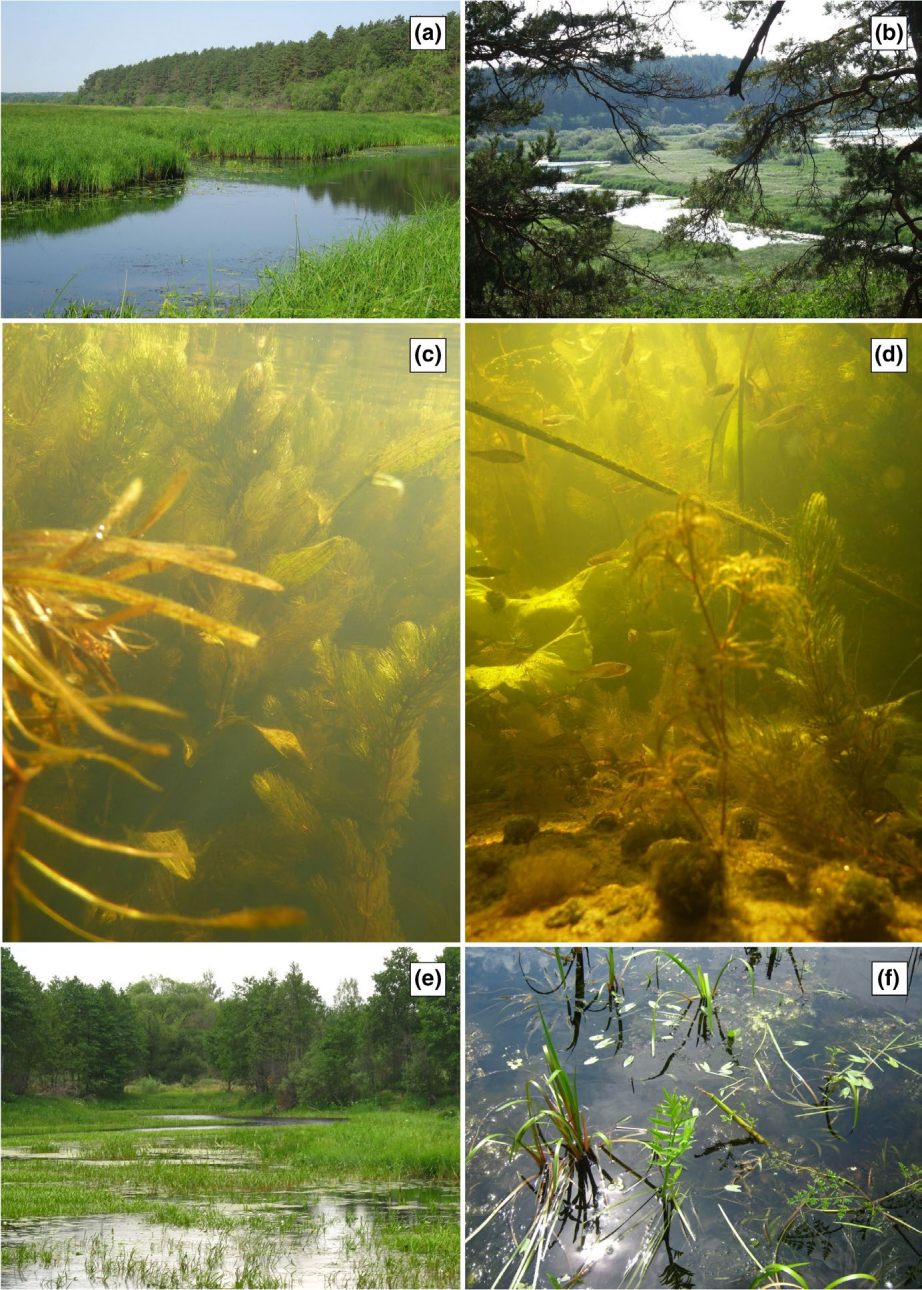
Habitat and view of *Placobdella costata*; a–d—site no 2, Lake Oxbow Dnieper Valley; e, f—view of site no 3, Lake Oxbow, Ptsich Valley

**FIGURE 3:**
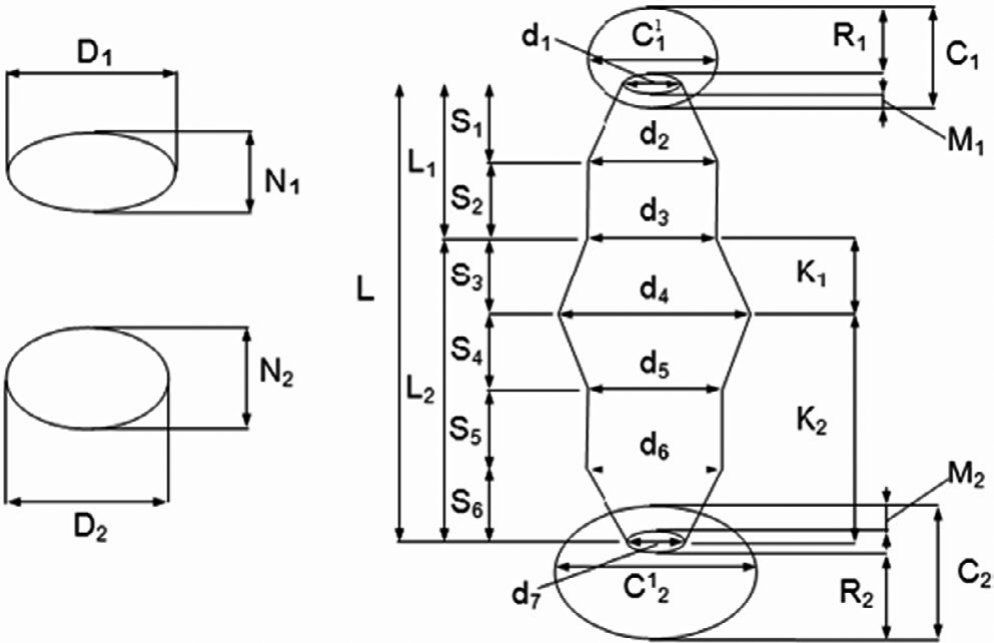
Model of the leech body form. The meaning of the symbols used is given in the text

The authors describe the results of their experiences from four locations in Central and Eastern Europe (Figure 1; Table 1). *Placobdella costata* (five adult and 44 juvenile specimens; Figure 2) and other species of the family Glossiphoniidae (coming from various places in Poland, some of them were described in the work of Bielecki et al., 2009) were measured based on the parameters of the body form model of leeches created by Bielecki and Epshtein (1994, 1995) (Figure 3). The model presents the leech body on a plane, as two ellipses (that represent suckers) and trapeziums situated between them (representing anterior body part—trachelosome—2 trapeziums; posterior body part—urosome—4 trapeziums). Besides, transverse sections through the trachelosome and urosome are considered as two ellipses.

**TABLE 1:** The events places characteristic

Site no.	Location	GPS coordinates	Date of observation	Characteristics of the bottom	Presence of turtles^a^	Birds/mammals presence^a^
1	Yaselda Valley (bel. Яceльдa), between Staramlyny (bel. Cтapaмлыны) and Sporava (bel. Cпopaвa), Belarus, S part (Polesie)	ca. 52° 23′ N, 25° 16′ E	August 2000	Sandy bottom densely covered with aquatic vegetation	No turtles were found but a habitat suitable for their occurrence and reproduction	Birds/moose
2	Lake Oxbow in the Dnieper Valley (called Lake Vir, bel. Bip) near Hlushets (bel. Глyшэц), 17 km to NNW of Rechytsa (bel. Pэчыцa), Belarus, S part (Polesie)	52° 30′ 02.5″ N, 30° 19′ 00″ E	July 2016	Sandy bottom densely covered with aquatic vegetation dominated by *Potamogeton obtusifolius* and *Ceratophyllum demersum*	No turtles were found but a habitat suitable for their occurrence and reproduction	Birds/moose
3	Lake Oxbow in the Ptsich Valley (bel. Пцiч), downstream of Slobodka (bel. Cлoбoдкa) village, 2.5 km to South of Kapatkyevichy (bel. Кaпaткeвiчы), Belarus, S part (Polesie)	52° 17′ 35.4″ N, 28° 48′ 54.8″ E	August 2017	Strongly overgrown with by floating patches of *Stratiotes aloides* and *Chara* beds	No turtles were found but a habitat suitable for their occurrence and reproduction	Birds/moose
4	Łyna River within the city (Olsztyn), Poland ‐ north‐eastern part (Masurian Lake District)	53° 46′ 25″ N, 20° 28′ 56″ E	May 2018	Sandy‐muddy bottom, covered with bur‐reed (*Sparganium* sp.)	No—the area is within the city and is heavily influenced by human pressure	Birds/beavers

^a^ Confirmed on the basis of literature data.

Based on 29 parameters of the morphometric description (Figure 3), 19 body proportions indexes are as follows:

- relative body length: L/D_2_ = ratio of length to largest width of urosome.
- anterior sucker: C_1_^1^/d_1_ = ratio of horizontal diameter of sucker to trachelosome width at sucker junction; C_1_^1^/D_1_ = ratio of horizontal diameter of sucker to the greatest width of trachelosome; R_1_/M_1_ = ratio of dorsal part of sucker to its ventral part; C_1_^1^/C_1_ = ratio of horizontal diameter of sucker to its vertical diameter.
- anterior body part (trachelosome): L_1_/D_1_ = ratio of trachelosome length to its greatest width; D_1_/N_1_ = ratio of the greatest trachelosome width to its greatest height; S_1_/S_2_ = index describing position of the greatest width of trachelosome.
- posterior body part (urosome): L_2_/D_2_ = ratio of urosome length to its greatest breadth; D_2_/N_2_ = ratio of the greatest urosome width to its greatest height; K_1_/K_2_ = ratio describing position of the greatest width of urosome.
- posterior sucker: C_1_^1^/d_7_ = ratio of horizontal diameter of sucker to urosome width at sucker junction; C_1_^1^/D_2_ = ratio of horizontal 16 diameter of sucker to the greatest body height; R_2_/M_2_ = ratio of dorsal part of sucker to its ventral part; C_1_^1^/C_2_ = ratio of horizontal diameter of sucker to its vertical diameter.
- relations between urosome and trachelosome: L_2_/L_1_ = ratio of urosome length to trachelosome length; D_2_/D_1_ = ratio of the greatest width of urosome to the greatest width of trachelosome; N_2_/N_1_ = ratio of the greatest height of urosome to the greatest height of trachelosome.
- proportions of suckers: C_2_^1^/C_1_^1^ = ratio of horizontal diameter of posterior sucker to horizontal diameter of anterior sucker.

Similarities of the body form were found with the use of the cluster analysis (Ward's method, Manhattan distance).

## 3 RESULTS

**FIGURE 4:**
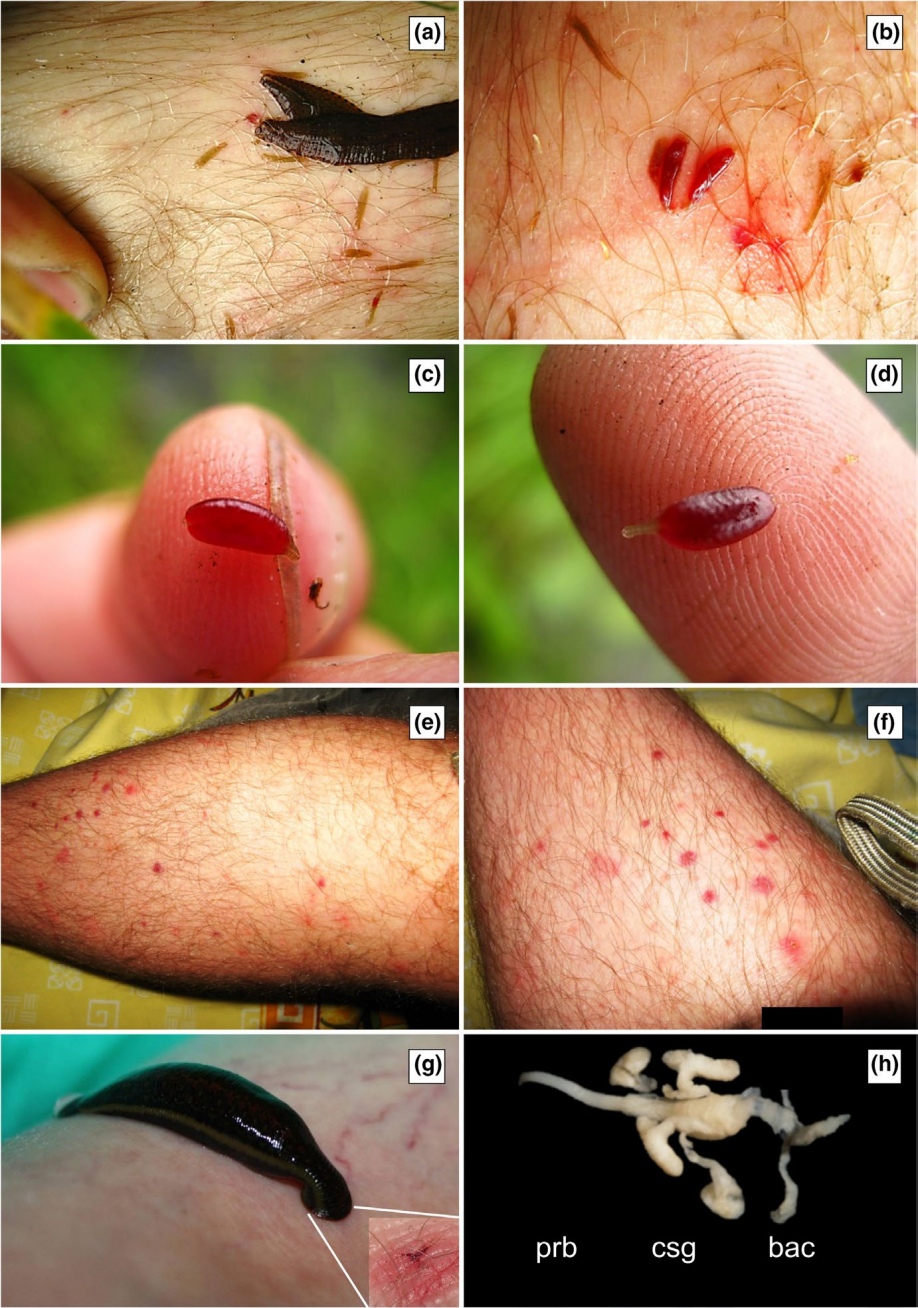
*Placobdella costata*. a—adults and juveniles, b—juveniles who have drunk blood and individuals who have not taken blood yet, c, d—juvenile specimens full of blood, visible division of the body into a wide urosome and narrow trachelosome, e, f—characteristic incision traces after blood sampling *P*. *costata*, small roundish, g—*H*. *verbana* and trace characteristic incision similar to the symbol Mercedes—*H*. *medicinalis*, h—proboscis with salivary glands, prb—proboscis, csg—compact salivary glands, bac—bacteriomes

In four locations in Europe, turtle leeches *Placobdella costata* attacking humans were observed by researchers taking hydrobiological samples (floristic and faunistic), without protective clothing. At site number 1, the Yaselda Valley, Belarus, three small juvenile individuals (2–2.5 cm) were observed on the leg in the calf area. At site number 2, Lake Oxbow of the Dnieper River in the Dnieper Valley, Belarus, two large, several‐centimeter long dark individuals and about 20 juveniles were present on one leg and five on the other leg (Figure 4a). Most of them were located under the knee of the right leg, approximately at the height of the upper part of the tibia, and single ones were lower, including the foot. On the leg of the second person, a single young individual was observed slightly above the ankle of the left leg.

Juveniles were transparent and difficult to see. Puncture of the skin was felt earlier and later in the form of tingling. Smaller leeches sucked blood briefly, only about 40 min to an hour. After feeding, leeches changed from small greenish‐yellow to red once they were filled with blood and from the form of threads to several times larger (Figure 4b–d). The parents, with the juvenile leeches attached to their ventral side, attacked the legs and first put the proboscis into the blood vessels, for example, in the hair follicle. When the blood began to flow into the wounds, the young individuals entered their proboscides. Juvenile leeches were also observed to pierce the skin on their own and be difficult to remove.

At site number 3 in Lake Oxbow in the Ptsich Valley, Belarus, two large, dark brown leeches and about 15 small ones were observed. Almost all of them were located on the one leg below the knee, including the arch of the foot. At site number 4 in the Łyna River channel, Poland, only a single adult specimen was observed to suck human blood from the right lower limb, at the ankle in the area of the Achilles tendon.

All of the abovementioned cases of blood sucking were accompanied by a tingling sensation, and the wound bleeding persisted for several hours after the leech had been removed. Red small circles with a hole in the center formed after the penetration of the proboscis and quickly became slightly itchy and red and then blue and purple. However, they were not bothersome and disappeared after about a week if they were not mechanically damaged through scratching (Figure 4e and f).

Due to the customs regulations, leeches observed in Belarus at sites 1–3 were left in the field. The specimen from site no. 4 in Poland was preserved in 70% ethanol and stored at the Department of Tourism, Recreation and Ecology, University of Warmia and Mazury in Olsztyn (Poland). The taxonomic identification of this leech was carried out on the basis of morphological and anatomical characters (Figure 4h).

### 3.1 Morphometric analysis

**FIGURE 5:**
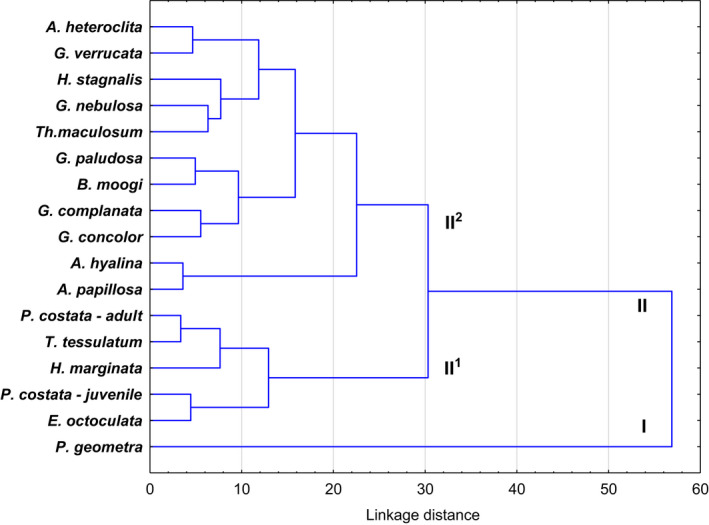
Tree diagram for 16 leech species based on mean value of 19 body index (Table 2). Ward's method, Manhattan distances

The algorithm for combining or grouping trees was chosen to interpret the similarity of the body form of 14 species from the Glossiphoniidae family and two species from the families *Piscicola geometra* and *Erpobdella octoculata*. The procedure allowed the species morphotypes to be divided into (Figure 5)—the monotype cluster (I), in which *P*. *geometra* was found, and the polytype cluster (II) consisting of the remaining species. The latter cluster was divided into subclusters II^1^ and II^2^ and the latter included juvenile *P*. *costata*, and adult *Hemiclepsis marginata*, *Theromyzon tessulatum*, and *P*. *costata*. In this subcluster, adult *P*. *costata* and juvenile both *T*. *tessulatum* and *P*. *costata* and *E*. *octoculata* have the most similar body forms. It follows that *P*. *costata* minors have “tape” type body form (similar to *E*. *octoculata*) which differs from adult *P*. *costata* with a “leaf” shape (Table 2).

**TABLE 2:** Mean value of 19 body indexes in 16 leech species

Species	L/D_2_	C_1_^1^/d_1_	C_1_^1^/D_1_	R_1_/M_1_	C_1_^1^/C_1_	L_1_/D_1_	D_1_/N_1_	S_1_/S_2_	L_2_/D_2_	D_2_/N_2_	K_1_/K_2_	C_2_^1^/d_7_	C_2_^1^/D_2_	R_2_/M_2_	C_2_^1^/C_2_	L_2_/L_1_	D_2_/D_1_	N_2_/N_1_	C_2_^1^/ C_1_^1^
*Glossiphonia complanata* (Linneaus, 1758)	1.1	2.0	0.2	0.7	1.1	0.6	1.7	1.3	0.7	2.0	0.4	2.5	0.3	0.7	1.0	1.8	1.5	1.3	1.8
*Glossiphonia concolor* (Apáthy, 1883)	1.4	1.7	0.2	1.0	1.1	0.9	3.0	1.1	0.8	3.2	0.5	2.5	0.3	1.1	0.9	1.4	1.5	1.4	1.9
*Glossiphonia nebulosa* Kalbe, 1964	1.7	0.6	0.3	2.8	0.7	0.9	0.9	1.0	1.1	2.8	0.3	0.6	0.3	0.5	1.0	2.0	1.6	1.2	1.8
*Glossiphonia paludosa* (Carena, 1824)	2.2	1.3	0.2	1.7	0.9	1.3	2.1	0.7	1.5	1.8	0.4	2.3	0.3	1.4	1.4	1.6	1.4	1.7	2.2
*Batracobdelloides moogi* Nessemn and Csanyi, 1995	2.3	2.7	0.2	1.7	1.2	1.2	1.7	1.5	1.4	1.9	0.4	2.3	0.3	1.3	0.9	1.6	1.3	1.2	1.7
*Hemiclepsis marginata* (O.F. Müller, 1774)	2.0	5.3	0.4	1.0	1.5	1.1	1.8	1.7	1.2	1.7	0.5	1.6	0.3	1.0	0.7	1.5	1.3	1.4	1.0
*Helobdella stagnalis* (Linneaus, 1758)	1.7	1.1	0.3	0.7	1.5	1.2	1.7	1.0	1.1	2.1	0.7	0.9	0.4	1.0	1.3	1.7	1.9	1.5	2.3
*Glossiphonia verrucata* (Fr. Müller, 1844)	1.3	2.5	0.3	0.5	1.8	0.7	1.1	0.9	0.9	1.4	0.4	1.1	0.1	1.4	0.9	2.2	1.7	1.3	0.6
*Alboglossiphonia papillosa* (Carena, 1820)	1.6	1.1	0.1	2.0	0.7	0.7	4.3	0.8	1.2	3.9	0.6	0.8	0.1	1.3	0.7	2.7	1.5	1.6	1.5
*Alboglossiphonia hyalina* (O.F. Müller, 1774)	1.8	0.9	0.1	2.0	0.7	0.8	5.0	0.9	1.1	4.4	0.4	0.8	0.1	1.3	0.7	1.9	1.4	1.5	1.6
*Alboglossiphonia heteroclita* (Lukin, 1976)	1.3	1.6	0.2	0.5	0.7	0.7	1.1	1.0	0.9	1.3	0.4	1.5	0.2	1.3	0.7	2.5	1.7	1.5	1.5
*Placobdella costata* (Fr. Müller, 1846)—adult	1.6	4.2	0.4	0.6	1.5	0.8	1.7	1.1	1.0	1.9	0.4	2.5	0.3	1.5	1.0	2.0	1.5	1.3	1.3
*Placobdella costata* (Fr. Müller, 1846)—juvenile	2.5	4.4	0.4	0.5	1.3	0.7	2.1	1.0	0.9	2.4	0.3	0.9	1.0	1.1	1.0	1.8	0.9	1.0	1.4
*Theromyzon tessulatum* (O.F. Müller, 1774)	1.5	4.3	0.4	0.5	1.5	1.0	1.7	1.4	0.9	2.0	0.4	3.3	0.3	1.8	1.1	1.5	1.7	1.5	1.2
*Theromyzon maculosum* (Rathke, 1862)	1.9	0.8	0.4	1.8	1.3	1.0	1.2	0.9	1.3	1.9	0.3	0.8	0.2	1.4	0.8	2.4	1.8	1.0	1.4
*Erpobdella octoculata* (Linnaeus, 1758)	4,4	4,6	0,4	0,6	1,2	0,9	2,2	0,9	1,2	2,3	0,4	0,7	0,8	1,3	1,1	1,7	1,1	1,2	1,3
*Piscicola geometra* (Linnaeus, 1761)	13,0	2,4	1,5	2,7	1,0	3,7	1,1	0,7	10,0	1,2	0,5	2,2	1,8	2,2	1,0	2,7	1,4	1,3	1,7

## 4 DISCUSSION

Turtle leech, the common name of *Placobdella costata*, emphasizes its close relationship with freshwater turtles and especially with the European pond turtle *Emys orbicularis*. Although the distribution area of the European pond turtle coincides to a large extent with data on the occurrence of *P*. *costata*, the presence of leeches in England, the Netherlands, and perhaps Finland does not confirm the host specificity of this species (Bielecki et al., 2012). When examining *P*. *costata* environments in which the European pond turtle does not occur, the following birds were among its supposed hosts: great crested grebe (*Podiceps cristatus* L.), mallard duck (*Anas platyrhynchos* L.) (Kufel, 1969), coot (*Fulica atra* L.) (Radkiewicz, 1972), and black stork (*Ciconia nigra* L.) (Matysiak, 1964). Elliott and Tullett (1982) explain the leech's presence in the British Isles because of the relationship with migrating birds whose flight routes run across continental Europe. Blood taken from bird species in laboratory conditions substantiates this thesis (Wilkialis, 1970). In the face of the emergence of a potential source of food and physiological possibilities of obtaining it, predatory leeches take advantage of this opportunity. This gives a chance of survival to leeches and is consistent with experimental observations, which describe much wider food spectra than those that are confirmed in field observations (Esbérard et al., 2005; Wilkialis, 1970, 1973, 1984). Other *Placobdella* species are also mainly ectoparasites on turtles but are able to attack animals other than reptiles, and both *Placobdella parasitica* (Say, 1824) and *Placobdella ornata* (Verrill, 1872) optionally attack humans (Jones & Woo, 1990; Moser et al., 2010; Oceguera‐Figueroa & Pacheco‐Chaves, 2012; Sawyer, 1986; Siddall & Bowerman, 2006; Siddall & Gaffney, 2004).

*Placobdella costata* has a number of features that indicate the possibility of a wider spectrum of hosts. Strong proboscis especially in the basal part, one of the most important, seems to give the leech the ability to puncture human skin coatings in any place (according to our observations: arch of the foot, ankle and knee area, calf), which results in a lack of preference for location within the lower limb. Compared with the *P*. *costata* proboscis, those of other such rhynchobdellid leeches as *Theromyzon maculosum* O.F. Müller (representative of “Rhynchobdellida” feeding on the blood of birds) viewed at the same magnification are almost invisible. The difference is so significant because *T*. *maculosum* parasites on birds and draws blood from their mucous membranes of the anterior digestive and respiratory systems (Bielecki et al., 2009).

However, traces of penetration of the human skin with the proboscis and the resulting changes on the skin were completely different from the traces of blood leech by *Hirudo medicinalis* L. or *H*. *verbana* Carena leech, which have jaws (Figure 4g) (Sawyer, 1986). *Placobdella costata* leaves more or less rounded traces, much smaller than the medical leech, who are bright red and have three distinct grooves resulting from the action of three jaws. For some hosts, members of genus *Placobdella* are vectors of hemogregarine and trypanosome blood parasites (Barta & Desser, 1989; Siddall & Desser, 1990, 1991, 192a, 1992b, 1993, 2001). The confirmation of blood‐sucking capacity from human by *P*. *costata* is important information in the context of human health.

Like other representatives of Glossiphoniidae, adult *Placobdella costata* care for embryos and their young (Bielecki et al., 2012; Wilkialis, 1970). This was also visible during our observations, when adults with attached juvenile specimens attacked the legs of the researcher and first took blood, which also flowed into the wounds. Some young leeches introduced the proboscis into these wounds and took blood (Figure 4a and b), and others including adults successfully pierced the skin. In Belarus, during two incidents, two to three adults and a “swarm” of the young were attacking. Such behavior in the care of the offspring is very helpful in the survival of young leeches.

Leeches from the family Glossiphoniidae are represented by the most numerous species (Sket & Trontelj, 2008) and have the largest number of hosts, from invertebrates to almost all vertebrates. Various nutrition methods as well as unique care for a large number of polylecytal eggs and offspring most likely ensured their evolutionary success (Bielecki et al., 2014; Kutschera & Wirtz, 2001). In addition, one species of Glossiphoniidae cares for the offspring of other species in this family (Wilkialis, 1970). While breeding *P*. *costata* and *T*. *maculosum* in one aquarium, the authors (unpublished data), similarly to Wilkialis (1970), observed the interesting phenomenon of parental care. It may be because both **s**pecies feed on the blood of vertebrates (birds and reptiles, former Gadocapidae Sauropsida). While the young *P*. *costata* leeches are sucking blood, their body shape changes. The body form of leeches (Hirudinida) was developed by Bielecki and Epstein (1994) as a geometric model that contain**s** twenty‐five measurements and their nineteen proportions (features). From this model, it follows that leeches have four body forms: “cylinder,” “tape,” “flask‐like,” and “leaf,” which is characteristic of leeches from the Glossiphoniidae family.

Young leeches have a different body form than adults. It is a flattened “cylinder” or “tape” (Figure 4a and b), which during blood collection changes into a deceptively body‐like form (“butt”) similar to leeches from the Piscicolidae family of the genus *Limnotrachelobdella* where it is pronounced trachelosoma and urosoma (Figure 3) (Bielecki, 1997; Cichocka & Bielecki, 2015; Cichocka et al., 2018). This body form probably occurs for a short period during the early ontogenesis of *P*. *costata*. Adult *Placobdella costata* no longer take this body form but have a “leaf” type (Bielecki, 1997; Bielecki et al., 2014; Cichocka & Bielecki, 2015). The body‐flattened “cylinder” form of young *P*. *costata* leeches even before the first blood sucking corresponds to the ancestor of leeches, which sucked blood and whose body shape resembled leeches from the families Piscicolidae and Erpobdellidae (Cichocka & Bielecki, 2015; Siddall, 2002; Siddall & Burreson, 1995, 1996, 1998). In our study, the morphometic analysis proved the similarity of the body form of young *P*. *costata* and representatives of *Erpobdella* (Figure 5). The eprobdellids, piscicolids, and juvenile *P*. *costata* have very similar main body parameters with only differences within the anterior sucker. Cichocka and Bielecki (2015) showed that on the leech body form model, the cladistic interpretation of leech evolution based on morphometry (LBF model) only slightly diverges from the picture of evolution based on a molecular level. In addition, these studies have shown that individual leech taxa at a family level have morphometric synapomorphy (invariants), and Glossiphoniidae have 12 of them. Such a synapomorphy in the Glossiphoniidae family is the ratio of body length to its greatest width and thickness and derivatives of these relationships (L/D_2_, L_2_/D_2_, K_1_/K_2_, N_2_/L_2_, D_2_/D_1_). These are features related to the care of the offspring, which in *P*. *costata* are revealed already at an early stage of ontogenesis, actually at the first blood suction (Bielecki & Epstein, 1994; Bielecki et al., 1999).

We are getting more and more information about leeches’ feeding relationships that tell**s** us how this process could look from the point of view of evolutionary phenomena. It allows us to hypothesize possible future hosts. This is important especially in light of the current global environmental changes occurring, accompanied by changes in the range of species occurrence of both parasites and their hosts. Our observations regarding *P*. *costata* nutrition can also be seen as an adaptation in the absence of a preferred food base. Moreover, the leech appear**s** to be much more mobile than its host since it is regularly found far outside the range of turtles (Vamberger & Trontelj, 2007), which might be explained by relation with such more mobile hosts as the beaver (*Castor fiber*), elk (*Alces alces*), and waterfowl (Biegel & Grosser, 2004; van Haaren et al., 2004).

The association of leeches with beavers may explain the presence of *P*. *costata* in the Łyna River (Olsztyn, Poland), where, unlike turtles, the presence of beavers was recorded (Grzybowski & Endler, 2012). Their activity is visible even in urban sections, where anthropogenic pressure is strongly marked. Despite the existence of potentially suitable habitats, the literature data and field observations do not indicate the presence of turtles and beavers at sites located in Belarus. It is worth paying attention to the numerous presences of waterfowl and the fact that the authors found the presence of elk in this area. The presence of elk was also confirmed in the vicinity of the Łyna River (Poland). The occurrence of leeches from the Glossiphoniidae family is associated with the presence of **s**uch stable environmental elements as aquatic vegetation (Adamiak‐Brud et al., 2018; Kubova & Schenkova, 2014; Kubova et al., 2013; Sawyer, 1986). *Placobdella costata* prefers places in the shallowest parts of water bodies with dense vegetation (Sapkarev, 1964), which are also suitable for feeding all of the abovementioned potential food sources, including the zone penetrated by humans (e.g., anglers). Our observations confirmed the presence of representatives of this species in the areas overgrown with submerged macrophytes, but no relationship with a specific species was found.

## 5 CONCLUSION

Our field observations confirm that juvenile forms of *P*. *costata* still under parental care are able to puncture the human skin and suck the blood. There was no preference for a specific place on the human limbs. We supplemented information on parental care strategies and morphological data about the body form of this species. Our analysis confirmed that the body form of leeches may change during their ontogeneses, which seems to be important in terms of species determination and classification. Based on our observations, we obtained more information about the potential role of mammals as hosts and dispersion vector of this leech. Our description, although it concerns one species, is universal and may be the basis for the consideration of broadening the spectrum of parasite hosts in the context of reducing biodiversity.

## CONFLICT OF INTEREST

The authors certify that they have no affiliations with or involvement in any organization or entity with any financial interest, or nonfinancial interest in the subject matter or materials discussed in this manuscript—no conflict of interest.

## AUTHOR CONTRIBUTIONS

**Joanna M. Cichocka:** Conceptualization (supporting); Supervision (supporting); Validation (lead); Visualization (equal); Writing‐original draft (supporting); Writing‐review & editing (supporting). **Aleksander Bielecki:** Conceptualization (lead); Supervision (lead); Writing‐original draft (equal); Writing‐review & editing (equal). **Izabela Jabłońska‐Barna:** Conceptualization (lead); Data curation (supporting); Project administration (equal); Validation (equal); Writing‐original draft (equal); Writing‐review & editing (equal). **Łukasz Krajewski:** Conceptualization (supporting); Investigation (equal); Visualization (lead); Writing‐original draft (equal); Writing‐review & editing (equal). **Katarzyna Topolska:** Data curation (supporting). **Joanna Hildebrand:** Writing‐review & editing (supporting). **Małgorzata Dmitryjuk:** Writing‐review & editing (supporting). **Anna Biedunkiewicz:** Writing‐review & editing (supporting). **Andrei Abramchuk:** Data curation (supporting); Investigation (supporting); Writing‐original draft (supporting).

## Data Availability

The authors agree to deposit their data in a public repository.
